# A Preliminary Investigation Regarding the Effect of Tennis Grunting: Does White Noise During a Tennis Shot Have a Negative Impact on Shot Perception?

**DOI:** 10.1371/journal.pone.0013148

**Published:** 2010-10-01

**Authors:** Scott Sinnett, Alan Kingstone

**Affiliations:** 1 Department of Psychology, University of Hawaii at Manoa, Honolulu, Hawaii, United States of America; 2 Department of Psychology, University of British Columbia, Vancouver, Canada; Duke University, United States of America

## Abstract

**Background:**

There is a growing chorus of critics who complain that many of the top-ranked professional tennis players who grunt when they hit the ball gain an unfair advantage because the sound of the grunt interferes with their opponent's game. However, there is no scientific evidence to support this claim.

**Methodology/Principal Findings:**

We explored this potential detrimental effect of grunting by presenting videos of a tennis player hitting a ball to either side of a tennis court; the shot either did, or did not, contain a brief sound that occurred at the same time as contact. The participants' task was to respond as quickly as possible, indicating whether the ball was being hit to the left- or right-side of the court. The results were unequivocal: The presence of an extraneous sound interfered with a participants' performance, making their responses both slower and less accurate.

**Conclusions/Significance:**

Our data suggest that a grunting player has a competitive edge on the professional tennis tour. The mechanism that underlies this effect is a topic for future investigation. Viable alternatives are discussed. For example, the possibility that the interfering auditory stimulus masks the sound of the ball being struck by the racket or it distracts an opponent's attention away from the sound of the ball.

## Introduction

Portuguese tennis had something to brag about last year. For the first time a Portuguese women's tennis player, Michelle Larcher de Brito, made it to the third round of the 2009 French Open. Unfortunately for Michelle she lost to Frenchwoman Aravane Rezai in a match where Michelle was heavily criticized for executing a loud and long grunt each time she hit the ball. The complaint is that Michelle, and many of the best players in tennis like her, such as Rafael Nadal, the Williams sisters, and Maria Sharapova (who grunts at over 100 decibels), may gain an unfair advantage by distracting their opponents with their grunts. Indeed, there is a growing chorus of critics who complain that many of the top-ranked professional tennis players are cheating when they grunt. This complaint has been voiced not only by the media and fans, but also by the athletes themselves [1], [2]. For instance, Martina Navratilova (former World number 1) recently said that grunting is “…cheating and it's got to stop” [1]. Navratilova's argument centered around the idea that it is important to hear the ball strike the racket, and that the sound of a grunt can mask or distract attention from this important moment. Accordingly, the governing body of the rules of tennis (International Tennis Federation, ITF) explicitly state (rule 26) that purposeful and excessive grunting is a hindrance and reason for a point penalty [3].

The importance of hearing the racket strike the ball dovetails with scientific multisensory research. For instance, laboratory research indicates that when two visual objects collide, the sound of that collision is critical to the perception of one item bouncing off the other [4]. Furthermore, it has also been shown that attention can be drawn to and focused on a visual event when it emits a sound [5]–[7]. Thus, it is possible that the sound provided by the grunt could interfere with these beneficial effects by either masking the sound of the racket hitting the ball, or by drawing attention away from this sound. Of course, it should be noted that a grunting player likely does not share the opinion that their grunt compromises an opponent's performance. Indeed, while there is good scientific evidence that performance on a visual task can be interfered with when a rare unexpected distracting sound occurs, such as a phone ringing during an exam [8], a predominant complaint is that tennis players grunt *too* frequently, so the grunts can hardly be unexpected. Moreover, the grunting players could even argue that their grunts, if anything, provide an additional and beneficial signal to their opponent regarding the force and the timing of the ball being struck.

There is clearly a need for some objective data on this matter. The present study took an initial step towards addressing this issue by using dynamic video based clips of a professional tennis player striking a ball with or without an accompanying auditory stimulus that occurred at the same time as the visual event of the ball being struck by the racket. Specifically, we presented videos of a tennis player executing a forehand or backhand groundstroke to the left or right side of the court. Critically, half of the videos included a superfluous sound whereas the other half did not. This approach offers an initial look at the current debate in tennis, that is, whether or not the sound of a grunt interferes with an opponent's performance. If grunting is detrimental to performance, then longer response latencies and/or higher error rates would be expected when participants judge the direction of a tennis shot that is accompanied by a sudden brief noise.

## Methods

### Ethics statement

Informed written consent, abiding to and approved by the University of British Columbia's Behavioural Research Ethics Board (BREB), was obtained prior to participating in the experiment. This study was approved by the University's BREB.

### Participants

Thirty-three undergraduate students from the University of British Columbia participated in exchange for course credit. None of the participants had more than recreational tennis experience. All reported normal hearing and normal or corrected-to-normal vision.

### Apparatus and stimuli

Participants sat approximately 60 cm from a computer screen in a dimly lit and sound attenuated testing room. The experiment was programmed and presented using DMDX software (http://www.u.arizona.edu/~jforster/dmdx.htm).

A total of 384 video clips were made of a professional tennis player hitting the ball (either forehand or backhand) to either the left or right of a video camera (Canon ZR10 digital video (DV) camera; 10× optical zoom, 200× digital zoom, image stabilizer, and 460K CCD pixel level) set up on the baseline of the court opposite the player. To be included as a video clip, the player had to hit the ball in a 2×2 meter target extending from the sideline and the baseline. The video clips were edited so as to include forehands hit crosscourt and down the line, and backhands hit crosscourt and down the line. There was a total of four clips for each shot type that were then edited such that each clip was played with or without a grunt, and ended either at contact (hard decision) or 100 ms after contact (easy decision). Each clip type (i.e., 32 total for each shot type, total of 128 video clips ranging in length from 1230 ms –1666 ms) was repeated three times for a total of 384 trials. As the sound of a grunt varies widely, we used a standard auditory stimulus —white noise (500 ms) that occurred during a tennis shot. Two loudspeakers were placed on either side of the computer screen that played the sound at a comfortable volume level (60 db). This stimulus configuration ensured that the auditory and visual events appeared to originate from the same spatial location. It should also be noted that because tennis grunts are generally far louder than our noise stimulus, the nonhuman auditory stimulus used here is an extremely conservative first-approximation of an actual tennis grunt.

### Procedure

Participants were required to respond as quickly and accurately as possible indicating the direction of the shot in each video clip (3 blocks of 128 separated by breaks for rest). They were required to use the M key on a keyboard with their right hand if they thought that the shot was going to their right, and the X on a keyboard with their left hand if they thought that the shot was going to their left. Each trial began with a fixation cross (1250 ms), followed by the video. The experiment lasted approximately 25 minutes.

## Results

**Figure 1:**
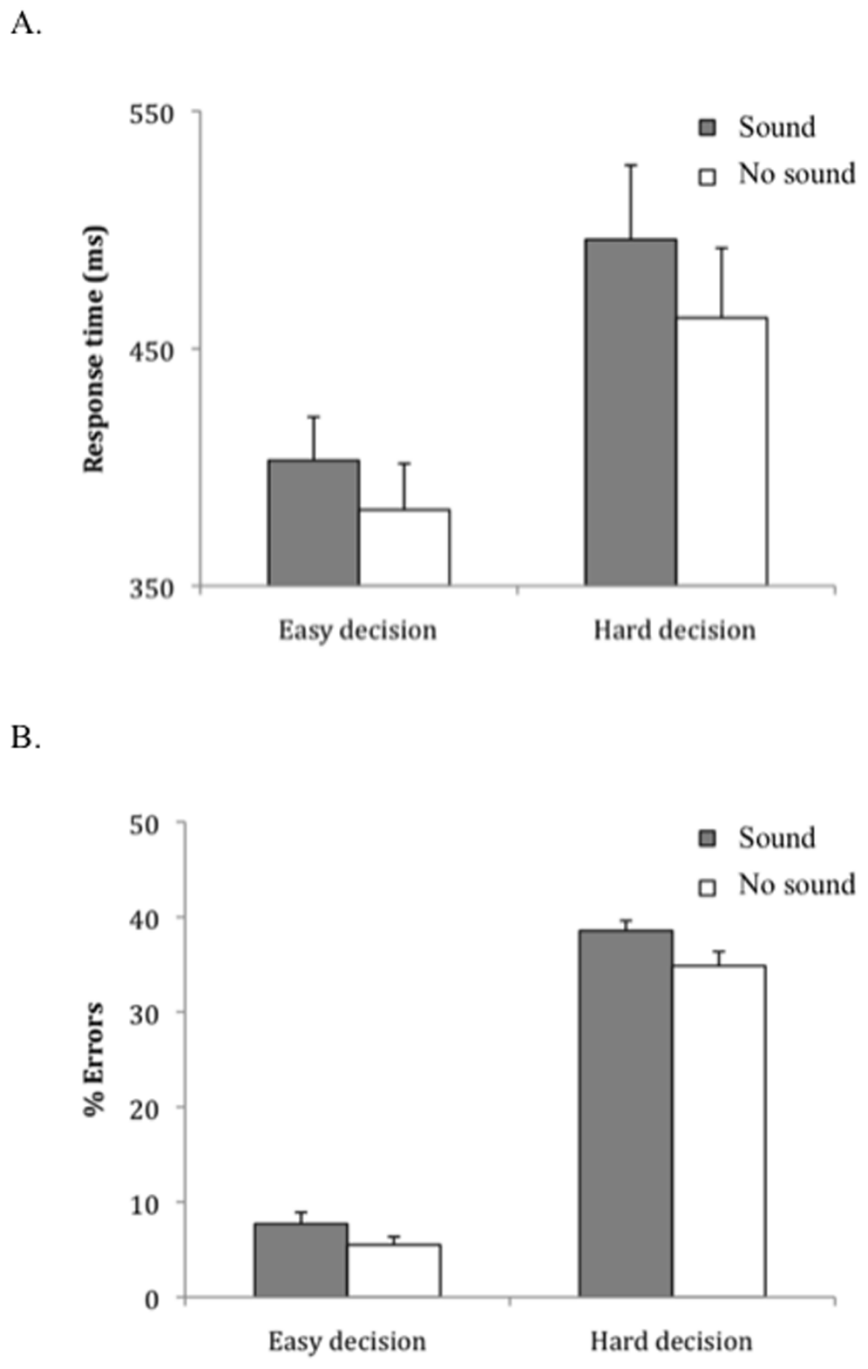
Response times and errors to easy- and hard-shot decisions when sound was or was not present. Dark grey bars represent when the sound was present and clear bars when the sound was absent for easy- and hard-shot decisions (A – response time and standard error bars in ms; B – total percentage and standard error bars of decision errors). All differences are significant.

**Figure 2:**
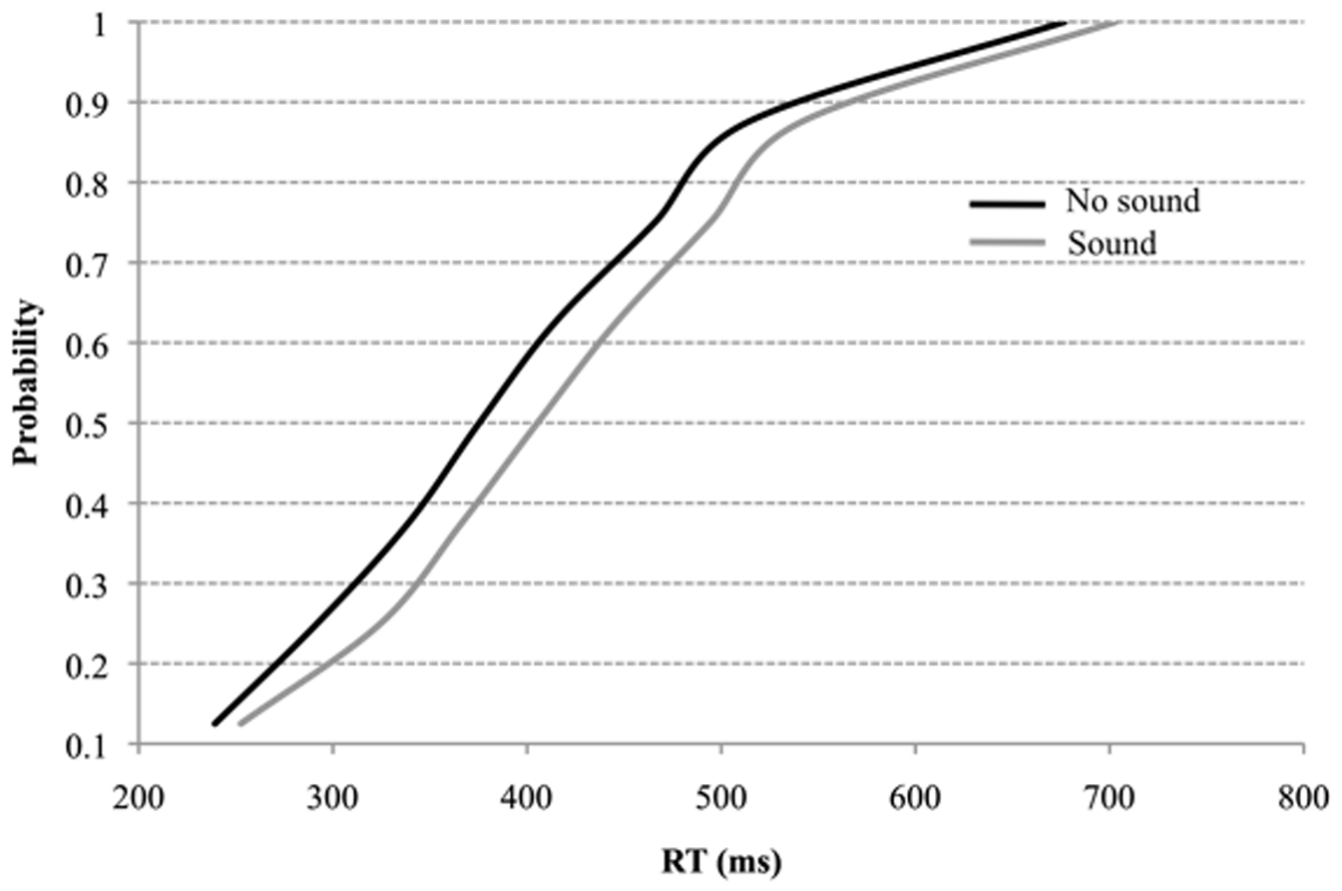
Cumulative probability distribution for response times pooled across decision type (hard and easy). Note that even the earliest RTs are affected when sound (grey line) is compared with no sound (black line) conditions.

Analyses of variance of the overall RT and error data with the within-subjects factors of Sound (Present vs. Absent) and Decision (Hard vs. Easy; i.e., clips ending at contact vs. clips ending after contact, respectively) were performed. The RT results revealed main effects of Sound and Decision, reflecting the fact that participants were slower to respond when a sound was present, F(1,32) = 31.1, p<.001, and the decision was hard, F(1,32) = 21.8, p<.001, but there was no interaction between sound and decision, F(1,32) = 1.74, p = .196. Similarly, for response accuracy, there were more errors for clips with the auditory stimulus F(1,32) = 16.0, p<.001, and hard decisions, F(1,32) = 525.8, p<.001, but no interaction, F<1. A second analysis was carried out after separating the data to include clips that ended at contact (Hard decision) and clips ending 100 ms after contact (Easy decision). When the sound was present and the video stopped at the time of contact, the participants were consistently 33 ms slower to respond to the direction of the ball (496 ms, SE = 32.2, versus 463 ms, SE = 30.2; t(32) = 3.7, p = .001), and they made 4% more decision errors (39%, SE = 1.1, vs. 35%, SE = 1.5; t(32) = 2.7, p = .012; see Figure 1). When the video ended 100 ms after contact the exact same pattern was observed. If the sound was present, participants were 21 ms slower to respond to the direction of the ball (403 ms, SE = 18.8, vs. 382 ms, SE = 20.3; t(32) = 3.7, p = .001), and they made 3% more errors (8%, SE = 1.2 vs. 5%, SE = .9; t(32) = 3.5, p = .001). Figure 2 shows the RT distributions for sound and no sound trials. It is clear that a sound is slowing even the shortest RTs, suggesting the negative impact of the noise stimulus is on the earliest perceptions of the tennis shot.

## Discussion

The main finding is clear-cut. When an additional sound occurs at the same time as when the ball is struck, participants are significantly slower (21–33 ms) and make significantly more decision errors (3–4%) regarding the direction of the ball both for easy and hard decisions alike. It is interesting to speculate how these values translate to the world of professional tennis. Despite serve speeds now frequently exceeding 100 mph [9], if one adopts a very conservative estimate that a professional tennis shot travels at 50 mph during a rally, a 21–33 ms response delay equates to a ball travelling two extra feet on every shot before an opponent can respond. This is a distinct advantage given that rallies on average last five to seven seconds, with opponents executing generally four directional changes per point with approximately three strokes per rally (the precise values will of course vary with factors like game strategy and court surface) [10]. Furthermore, based on data focusing exclusively on 481 matches played at Wimbledon from 1992–1995, an average of 6.4 points played per game can be calculated [11]. Therefore, between the average number of points played per game and the average number of strokes per point, the additional 3–4% errors observed here could be equivalent to an opponent being wrong footed by a grunting tennis shot nearly once every game. Given that only four points are required to win a game, this is potentially a tremendous advantage.

The aim of the present study was to determine if it is reasonable to conclude that a tennis grunt interferes with an opponent's performance. Our study shows that both response time and accuracy are negatively affected. With these facts in hand, the next question is to determine why, exactly, it has a detrimental effect on performance. If the experts are to be believed, the sound of the racket hitting the ball enables one to better judge the direction, spin, and velocity of the ball. In the professional tennis world, Navratilova has suggested that a grunt may *block* an opponent's ability to hear the sound that is made when the racket strikes the ball. However, this is not the only explanation for why a grunt may be detrimental. An alternative account is that one can still physically hear the sound of the ball hitting the racket, but the grunt draws *auditory* attention away from the sound of the ball and toward the sound of the grunt. A third possibility is that the grunt draws *visual* attention away from processing the visual event of a ball leaving the racket. An approximate equivalent of this would be how a cell phone conversation interferes with one's ability to attend to traffic on the road, i.e., one looks at the traffic but does not see it correctly [12]. On this score it is noteworthy that when we monitored the eye movements of a separate group of participants (n = 12) in the same task as the one reported here, all aspects of their eye movements (e.g., fixation number, latency and amplitude) were the same regardless of whether the additional sound was played or not. Thus, if the detrimental effect is on visual attention, it concerns covert visual attention (i.e., attention that does not involve concomitant changes in the position of the eyes [13]). We are currently manipulating systematically the time of the noise event and the moment that the ball strikes the racket; benchmarking the data against past studies of unisensory processing and multisensory integration. In doing so we will be able to tease apart whether the detrimental effect of the noise is due to auditory masking or attentional shifts within or between modalities.

An additional avenue for future research is to manipulate the expertise of the observer and use the sound of an actual tennis grunt. The former idea is of interest, as the participants in the present experiment were recruited so as to ensure that they were not professional tennis players. This was important so as to not limit the findings to only expert tennis players. Nevertheless, it is possible that the professional tennis players have unique strategies that may circumvent the negative impact of a grunt. For instance, prolific grunter Serena Williams has said that an opponent's grunt does not affect her, as she is concentrating on her own game [14]. However, given the self-reports from other professional tennis players that an opponent's grunting interferes with their play [1], [2], and our own data showing that an extraneous noise has a significant and negative impact on both response latency and accuracy, regardless of decision difficulty, it is reasonable to think that the negative effect of grunting persists for expert tennis players. Indeed, current research suggests that many multisensory phenomena are highly resistant to top-down processes [15] as integration occurs very early during low-level perceptual processing. This appears to be operating in our present study, as Figure 2 shows clearly that the earliest RTs are affected by the introduction of the noise stimulus.

Finally, it should be noted that while we chose to use generic white noise rather than a particular grunt, so as to control for extraneous differences between the grunts of individual tennis players (e.g., grunt length and/or intensity), it would be interesting to determine if different grunts vary in their affect on performance. These data will provide further insight into the mechanism that underlies a grunt's negative effect. For instance, it is reasonable that if a grunt distracts an opponent's attention, then the present results may in fact underestimate the negative effects of tennis-grunting as our nonhuman white noise stimulus was far more uniform and quieter than the grunts of a tennis player. This proposal is supported further by the well-established notion that people are especially tuned to attend to other humans, both in the visual [16] and auditory [17] domains.

It still remains unknown, and it will be very difficult to ascertain, whether many of the most prolific grunters intentionally grunt to interfere with their opponent's performance. Regardless, our data suggest that when they grunt they are gaining an unfair advantage. Our study indicates that grunting not only decreases an opponent's ability to judge the direction of a shot, it also reduces the amount of time they have to respond to every shot. These consequences on faster tennis surfaces, such as the grass courts of Wimbledon, or the hard courts of the Australian and US Open, are likely to be profound.
